# Characterization of a frozen shoulder model using immobilization in rats

**DOI:** 10.1186/s13018-016-0493-8

**Published:** 2016-12-08

**Authors:** Du Hwan Kim, Kil-Ho Lee, Yun-Mee Lho, Eunyoung Ha, Ilseon Hwang, Kwang-Soon Song, Chul-Hyun Cho

**Affiliations:** 1 Pain Research Center, Department of Orthopedic Surgery, Dongsan Medical Center, School of Medicine, Keimyung University, 56 Dalseong-ro, Jung-gu, Daegu, 41931 South Korea; 2 Department of Rehabilitation Medicine, Dongsan Medical Center, School of Medicine, Keimyung University, Daegu, South Korea; 3 Department of Orthopedic Surgery, Keunmadi Hospital, Kyungju, South Korea; 4 Department of Biochemistry, School of Medicine, Keimyung University, Daegu, South Korea; 5 Department of Pathology, Dongsan Medical Center, School of Medicine, Keimyung University, Daegu, South Korea

**Keywords:** Frozen shoulder, Animal model, Rat, Immobilization

## Abstract

**Background:**

The objective of this study was to investigate serial changes for histology of joint capsule and range of motion of the glenohumeral joint after immobilization in rats. We hypothesized that a rat shoulder contracture model using immobilization would be capable of producing effects on the glenohumeral joint similar to those seen in patients with frozen shoulder.

**Methods:**

Sixty-four Sprague-Dawley rats were randomly divided into one control group (*n* = 8) and seven immobilization groups (*n* = 8 per group) that were immobilized with molding plaster for 3 days, or for 1, 2, 3, 4, 5, or 6 weeks. At each time point, eight rats were euthanized for histologic evaluation of the axillary recess and for measurement of the abduction angle.

**Results:**

Infiltration of inflammatory cells was found in the synovial tissue until 2 weeks after immobilization. However, inflammatory cells were diminished and fibrosis was dominantly observed in the synovium and subsynovial tissue 3 weeks after immobilization. From 1 week after immobilization, the abduction angle of all immobilization groups at each time point was significantly lower than that of the control group.

**Conclusions:**

Our study demonstrated that a rat frozen shoulder model using immobilization generates the pathophysiologic process of inflammation leading to fibrosis on the glenohumeral joint similar to that seen in patients with frozen shoulder. This model was attained within 3 weeks after immobilization. It may serve as a useful tool to investigate pathogenesis at the molecular level and identify potential target genes that are involved in the development of frozen shoulder.

## Background

Frozen shoulder is a common shoulder disorder characterized by pain and gradual loss of active and passive glenohumeral motion that occurs in 2–5% of the general population [1–4]. However, the etiology and pathophysiologic mechanisms that lead to the development of frozen shoulder are poorly understood, and there is no consensus regarding optimal treatment [2, 4–7].

The primary pathologic site of frozen shoulder is the glenohumeral capsular tissue that particularly localized to the rotator interval and axillary recess [1, 6, 8]. Most likely, a sequential pathologic process from synovial inflammation to capsular fibrosis is the main source of pain and limited motion in frozen shoulder [2, 3, 5, 9–12]. Rodeo et al. [3] reported that a hypervascular synovial hyperplasia was present in the early stages, and resulted in eventual fibrosis in the subsynovium and capsule.

Recently, in vitro studies using specimens from patients with frozen shoulder have been focused on determining both an immunologic basis for the condition and the role of cell signaling and inflammatory mediators in its development [1–3, 5, 9–11, 13]. Despite intensive efforts, however, the underlying pathophysiologic process of frozen shoulder is not yet fully elucidated. Therefore, the development of animal models of frozen shoulder would seem to be essential in order to further characterize the underlying pathophysiology of frozen shoulder and the subsequent development of relevant therapeutic targets [14].

Although animal frozen shoulder models using mouse, rat, or canine have been developed [14–20], a rat shoulder contracture model using immobilization has been among the most frequently established due to similarities with human anatomy and a high levels of reproducibility [15–17, 20]. However, few studies have been conducted to investigate the serial pathophysiologic changes to the glenohumeral joint following immobilization while developing such animal frozen shoulder models. Therefore, generation and characterization of an animal frozen shoulder model would seem necessary to investigate the etiology and controlling mechanism at the molecular level in the pathogenesis of frozen shoulder.

The current study was conducted to investigate serial changes for histology of joint capsule and range of motion of the glenohumeral joint after immobilization in rats. We hypothesized that a rat shoulder contracture model using immobilization would be capable of producing effects on the glenohumeral joint similar to those seen in patients with frozen shoulder.

## Methods

The current study was performed under an experimental protocol approved by our Institutional Animal Care and Use Committee (No. KM 2014-48R1).

### Development of the animal model

**Fig. 1:**
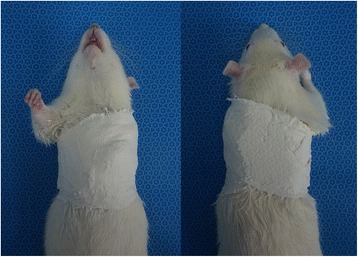
Immobilization is accomplished with applying molding plaster around the left whole arm including the shoulder and thorax of rats

Sixty-four 7-week-old male Sprague-Dawley rats (200–220 g) were randomly divided into one control group (*n* = 8) and seven immobilization groups (*n* = 8 per group). In the immobilization groups, rats were immobilized with molding plaster for 3 days, or for 1, 2, 3, 4, 5, or 6 weeks. Immobilization procedures were conducted under intraperitoneal anesthesia. Rats were anesthetized with an intraperitoneal injection of a mixture of tiletamine (25 mg/kg), zolazepam (25 mg/kg), and xylazine (0.5 mg/kg). Immobilization was successfully accomplished by applying molding plaster around the entire left arm including the shoulder and thorax (Fig. 1). The shoulder was fully adducted and internally rotated, and the elbow was flexed and pronated. To avoid complications associated with the use of molding plaster, such as loosening or breaking, an appropriate molding technique was devised through practice and a pilot study. The procedure was well-tolerated, and the rats were able to walk, self-feed, and survive for 6 weeks. There were no complications observed following the immobilization using molding plaster. At each time point, eight rats were euthanized for histologic evaluation of the axillary recess and measurement of the abduction angle.

### Measurement of abduction angles

**Fig. 2:**
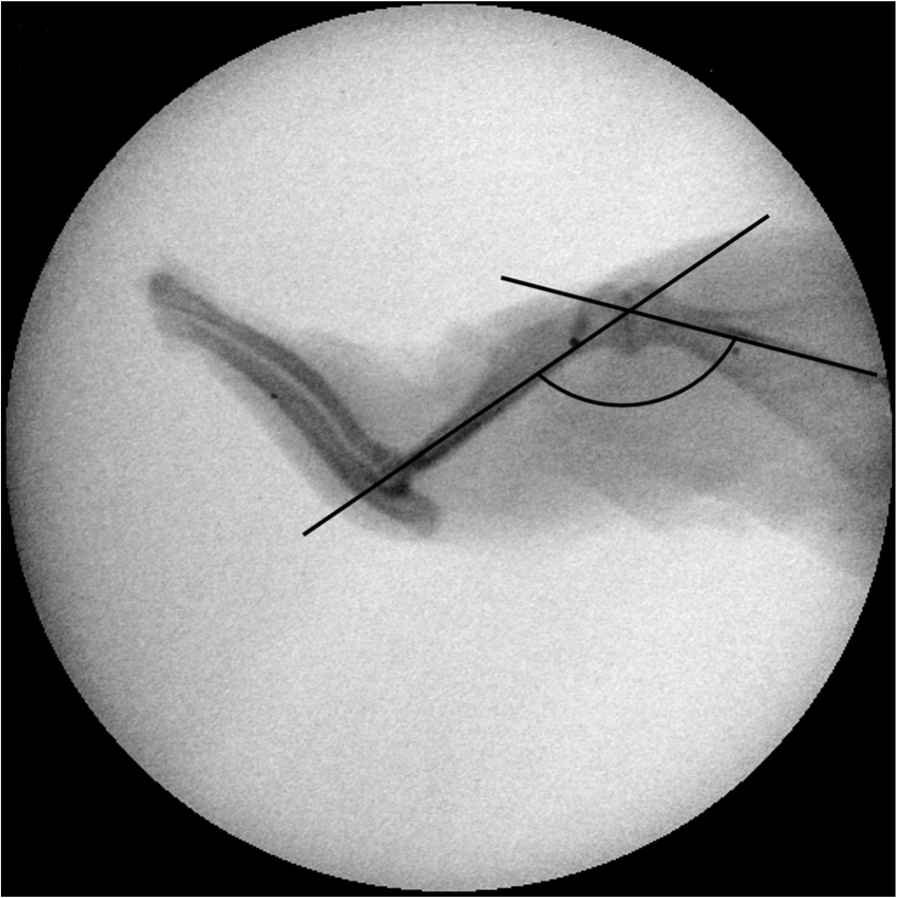
The abduction angle formed from the intersection of a line on the scapular spine and a line originating from the center of the humeral head to that of the humeral condyle is measured

After the shoulder girdle was removed from the trunk, abduction angles were measured using a radiograph taken in the maximal passive abduction, applied at a constant torque of 10-gram weight (3.92 × 10^−3^ N∙m torque) on the humeral shaft. To assess the abduction angles, the angle formed from the intersection of a line on the scapular spine and a line originating from the center of the humeral head to that of the humeral condyle was measured using Image J software (National Institutes of Health, Bethesda, MD, USA) (Fig. 2).

### Histology assessment

After the shoulder girdle was removed from the trunk, the specimens were processed in a standard manner. They were fixed in 10% formalin and decalcified with 10% ethylenediamine tetra-acetic acid. After decalcification, the specimens were embedded in paraffin. Standardized 2-μm thickness sections were obtained and stained with hematoxylin and eosin (H&E). To enhance the detection of fibrosis, the sections were stained with Masson’s trichrome.

### Statistical analysis

Statistical analyses were conducted using SPSS 18.0 software for Windows (SPSS Inc, Chicago, IL, USA). A repeated-measures analysis of variance (ANOVA) was used to identify the time effect on the abduction angle through 6 weeks. If the repeated measures ANOVA demonstrated a statistically significant time, multiple comparisons result was performed by contrast as Bonferroni correction. Paired *t* test was used to determine the difference between immobilized and non-immobilized shoulders. A *P* value <0.05 was considered statistically significant.

## Results

### Measurement of abduction angles

A significant difference in the abduction angle between immobilized and non-immobilized shoulders was observed beginning at 1 week after immobilization (Table 1). From that time point, the abduction angle at each subsequent time point in the immobilization groups was significantly lower than that in control group (*P* < 0.001). The nadir of the abduction angle was observed at 2 and 3 weeks after immobilization.

**Table 1:** Changes of the abduction angle

	Left (immobilized)	Right (non-immobilized)	*P* value^a^ (side difference)	Side difference	Time effect^b^ (multiple comparison)^c^
Control (C)	154.4° ± 5.0°	155.0° ± 5.3°	n.s.	5.0° ± 3.8°	*P* = 0.029 (C, D3 < W1,W2,W3,W4,W5,W6 and W1 < W2,W3)
Day 3 (D3)	153.8° ± 3.3°	154.8° ± 2.4°	n.s.	1.9° ± 1.2°	
Week 1 (W1)	123.1° ± 5.3°	157.5° ± 4.6°	<0.001	34.4° ± 5.6°	
Week 2 (W2)	103.1° ± 8.0°	158.1° ± 4.6°	<0.001	55.0° ± 6.5°	
Week 3 (W3)	97.5° ± 9.3°	153.1° ± 6.5°	<0.001	55.6° ± 7.7°	
Week 4 (W4)	105.6° ± 11.5°	153.1° ± 7.0°	<0.001	47.5° ± 12.2°	
Week 5 (W5)	105.0° ± 8.5°	155.0° ± 2.7°	<0.001	50.0° ± 8.0°	
Week 6 (W6)	99.7° ± 13.0°	150.6° ± 3.2°	<0.001	50.8° ± 11.8°	

*n.s.* not significant

^a^Paired *t* test

^b^repeated-measures one-factor analysis for time effect

^c^Multiple comparison by contrast

### Histologic findings

**Fig. 3:**
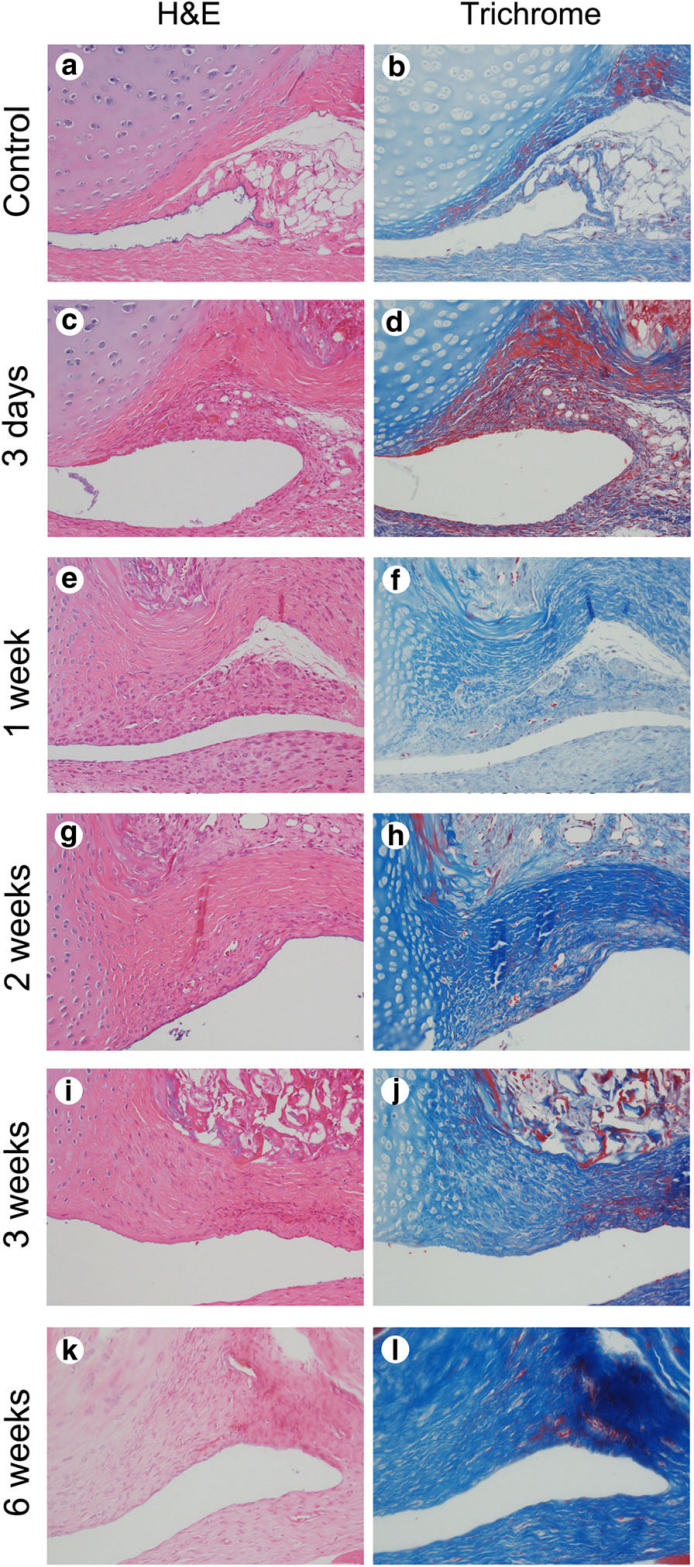
Serial microscopic findings of axillary recess of the glenohumeral joint in the control and immobilization groups. **a**, **b** Microscopic findings show no inflammation or fibrosis in the control group (H&E and Trichrome, ×200). **c**, **d** Inflammation with mild fibrosis is identified at 3 days after immobilization (H&E and Trichrome, ×200). **e**, **f** Fibrosis with mild inflammation is identified at 1 week after immobilization (H&E and Trichrome, ×200). **g**, **h** Fibrosis with minimal inflammation is identified at 2 weeks after immobilization (H&E and Trichrome, ×200). **i**, **j** Fibrosis with minute inflammation is identified at 3 weeks after immobilization (H&E and Trichrome, ×200). **k**, **l** Fibrosis without inflammation is identified at 6 weeks after immobilization (H&E and Trichrome, ×200)

The serial histologic evaluation revealed that the synovium and subsynovial structure of the axillary recess changed as a result of the procedure. The most noticeable changes observed in immobilized shoulders were the disappearance of the synovial fold and subsynovial fat tissue, capsular thickening, and capsular adherence to bony cortex. The decrease of synovial fold and subsynovial fat tissue, infiltration of inflammatory cells, proliferation of capillaries in the subsynovial tissue, and capsular thickening could be seen at just 3 days after immobilization. At 1 week after immobilization, the subsynovial fat tissue had almost disappeared and the capsular thickening tended to be distinct. At 2 weeks after immobilization, the synovium and subsynovial tissue of the humeral side adhered to bony cortex. Up until 2 weeks after immobilization, the infiltration of inflammatory cells was found in the synovial tissue. However, the inflammatory cells were diminished and fibrosis was dominantly observed in the synovium and subsynovial tissue at 3 weeks after immobilization. The histologic findings thereafter were similar to those observed at 3 weeks after immobilization. Masson's trichrome stain also showed that fibrosis had started at 3 days, and matured at 3 weeks after immobilization, respectively (Fig. 3). There were no changes to the synovium or subsynovial tissue observed in the non-immobilized side or in the control group.

## Discussion

The results of the current study demonstrated that a rat frozen shoulder contracture model using immobilization was capable of producing the pathophysiologic process of inflammation leading to fibrosis in the glenohumeral joint similar to that seen in patients with frozen shoulder. We found that this experimental model was accomplished within 3 weeks after immobilization.

The current state of knowledge reflects a poor understanding of the etiology and pathologic mechanism for frozen shoulder [7, 8, 14]. Histologically, Ozaki et al. [6] and Bunker et al. [13] reported that fibrosis of the capsule was the main lesion, but neither inflammation nor synovitis was observed [15]. However, the results from several studies indicate that inflammation in the synovium and fibrosis occurred together in the capsule [1–3, 9, 10]. Inflammation with hypervascular synovial proliferation and fibrosis of the joint capsule was already a well-known macroscopic and histological feature of frozen shoulder. To elucidate the exact etiology and pathophysiologic process of frozen shoulder, researchers recognized the necessity for animal models of this condition.

To date, seven studies describing the development of animal frozen shoulder models using canine, rat, or mouse have been reported [14–20]. The optimal choice of animal and methodology that would be most useful in creating a model for frozen shoulder remains unclear. Because the gross anatomy of the rat shoulder is quite similar to that of humans, four of seven of the aforementioned studies used rats as a frozen shoulder model [15–17, 20]. At present, the development of an animal model for primary frozen shoulder is considered to be an impossibility. Immobilization has been accepted as common predisposing factor for secondary frozen shoulder. Therefore, most studies have used immobilization for developing a secondary frozen shoulder model and demonstrated that shoulder immobilization can induce adhesion of the joint and capsular contracture [15–17, 20]. There are several different methods that can be used to achieve a shoulder contracture model using immobilization. Molding plaster or extra-articular fixation using a plastic plate or suture material has been used for this purpose [15–17, 20]. In the current study, we chose a rat shoulder contracture model using molding plaster because it was considered a simple and effective secondary frozen shoulder model based on literature reviews and the results of a pilot study.

Few studies have been conducted to determine when and how to change histology of the joint capsule and the range of motion of the glenohumeral joint during the development of animal frozen shoulder model. In the canine model study by Schollmeier et al. [19], the cellularity of the synovium and the number of subsynovial vascular channels were increased by as early as the fourth week after immobilization. At 8 weeks after immobilization, foci of adhesions began to appear between the adjacent synovial surfaces and the restricted range of motions occurred [19]. Villa-Camacho et al. [20] reported that extra-articular internal fixation of the glenohumeral joint during 8 weeks in a rat model induced a reduction in total range of motion. They concluded that this model accurately mimicked the pathologic changes of the joint capsule, as well as the glenohumeral kinematics that are characteristic of frozen shoulder, although this model did not replicate the initial inflammatory insult of frozen shoulder. In the rat model study conducted by Liu et al. [16], immobilization of the rat shoulder induced synovial hyperplasia of the joint capsule, adhesion of the subscapular bursa, and an increase of the capsular content of types I and III collagen. Additionally, they reported that the hyperplastic synovium of the anterior capsule obstructed the communication between the subscapular bursa and the glenohumeral joint cavity at 2 and 3 weeks [16]. Adhesion of the subscapular bursa appeared at 3 and 4 weeks, but no evidence of any hyperplasia of the capsule was found at 4 weeks [16]. They noted that the main pathological appearance in the early stage of immobilization was synovial hyperplasia of the capsule [16]. However, they did not mention the infiltration of inflammatory cells in the synovial tissue.

The experimental model employed in the current study revealed that the infiltration of inflammatory cells was found in synovial tissue until 2 weeks after immobilization. However, the inflammatory cells were diminished and fibrosis was dominantly observed in synovium and subsynovial tissue at 3 weeks after immobilization. The histologic findings thereafter were similar to those at 3 weeks after immobilization. The abduction angle was decreased from 1 week after immobilization. The nadir of abduction angle was observed at 2 and 3 weeks after immobilization. These results confirmed that the serial histologic changes on the glenohumeral joint after immobilization correlated with the restricted range of motion of the glenohumeral joint in a rat frozen shoulder model. Considering these findings, the interval from 3 days to 2 weeks after immobilization may be involved in the pre-adhesive and freezing phases of human frozen shoulder with dominant inflammation [21]. The period after 3 weeks may be involved in the frozen phase with dominant fibrosis [21]. These findings showed that rat shoulder immobilization model reproduce similar pathophysiologic process of inflammation resulting in fibrosis on the glenohumeral joint as is seen in patients with frozen shoulder. Their results also seemed to explain the heterogenous clinical manifestations of primary and secondary frozen shoulder. Additionally, the rat frozen shoulder model using immobilization assessed in this study was shown to be achievable 3 weeks after immobilization.

Although the rat frozen shoulder model using molding plaster employed in the current study requires practice and skill attained through a pilot study. Once mastered, it has been found to be highly reliable and reproducible. The rats tolerated immobilization by molding plaster during a 6-week period without any major issues or complications. We did not find any noticeable changes in the activity level or eating habits at the time of euthanasia. Therefore, the present model may serve as a useful tool to investigate pathogenesis at the molecular level and identify potential target genes that are involved in the development of frozen shoulder. Further study will be needed to evaluate gene expression analysis of the joint capsule in rat frozen shoulder models.

There were several limitations to our study. First, the rat shoulder may differ from the human shoulder because a rat is a quadruped animal and its shoulder is a weight-bearing joint. Second, there are some discrepancies between this model and human frozen shoulder. This is likely due to the use of a secondary contracture model after immobilization. However, to the best of our knowledge, ours is the first study to investigate the serial changes for histology of the axillary recess capsule and range of motion of the glenohumeral joint after immobilization in rats. Third, we did not investigate any changes after remobilization on the glenohumeral joint during the development of animal model for frozen shoulder.

## Conclusions

Our study demonstrated that a rat frozen shoulder model using immobilization generates the pathophysiologic process of inflammation leading to fibrosis on the glenohumeral joint similar to that seen in patients with frozen shoulder. This model was attained within 3 weeks after immobilization.
